# Evaluating the effects of evidence-based nursing on length of hospital stay, duration of mechanical ventilation, symptom relief, and complication rates in children with severe adenoviral pneumonia: a prospective randomized controlled trial

**DOI:** 10.1590/S1678-9946202567013

**Published:** 2025-02-17

**Authors:** Shali Wu, Sha Zhu, Hui Wen, Tuhong Yang, Yazi Liu, Ying Peng

**Affiliations:** 1 Hunan Normal University, Hunan Provincial People’s Hospital, Department of Neonatology, ChangSha, Hunan, China; 2 Hunan Normal University, Hunan Provincial People’s Hospital, ChangSha, Hunan, China; 3 Hunan Normal University, Hunan Provincial People’s Hospital, Department of Child Respiratory, ChangSha, Hunan, China; 4 Hunan Normal University, Hunan Provincial People’s Hospital, Child Intensive Care Unit, ChangSha, Hunan, China

**Keywords:** Evidence-based, Nursing care, Mechanical ventilation, Children, Adenoviral pneumonia

## Abstract

We conducted a prospective randomized controlled trial to evaluate the effect of evidence-based nursing care on length of hospital stay, duration of mechanical ventilation, symptom relief, and complication rates among mechanically ventilated children with severe adenovirus pneumonia. A total of 257 children admitted to Hunan Provincial People’s Hospital in Changsha from February 2018 to December 2021 were enrolled. Two patients withdrew from the study, resulting in 124 cases in the conventional care group and 131 cases in the evidence-based care group. Primary outcomes included time to resolution of signs and symptoms, length of hospital stay, complication rates. Secondary outcomes were blood biomarker levels and successful weaning results. The evidence-based care group demonstrated significantly higher overall efficiency than the conventional care group (98.47% vs. 95.97%, p<0.05). Additionally, the evidence-based care group demonstrated quicker resolution of cough, sputum, pulmonary rales, and fever, shorter hospital stays, and reduced need for mechanical ventilation (p < 0.05). The evidence-based care group had a significantly lower complication rate than the conventional care group (9.16% vs. 25.00%, p < 0.05). Post-care blood biomarker analysis showed decreased levels of leukocytes, calcitonin, and C-reactive protein in the evidence-based care group compared to the conventional care group (p<0.05). Evidence-based nursing interventions can improve outcomes for children with adenovirus pneumonia by reducing comorbidities, improving blood gas levels, reducing inflammatory responses, and improving the weaning success rate of mechanically ventilated children with severe adenoviral pneumonia.

## INTRODUCTION

Respiratory infections are a leading cause of childhood disease worldwide. These infections pose a significant burden to global health, surpassing other causes of disease^1^. According to Lancet, about 7.6 million children under five years old died worldwide in 2018, with infectious diseases accounting for 64% of these deaths. Among these, pneumonia was the most prevalent, claiming the lives of approximately 18% of children under five years^2^. Adenovirus (ADV) is a common cause of acute respiratory infections in children, accounting for 2–5% of respiratory diseases and 4–10% of childhood pneumonia cases^3^. This non-enveloped double-stranded DNA virus consists of approximately 69 types and seven species (A-G)^4-6^, each leading to different symptoms^7^. In recent years, ADV infections have become increasingly prevalent in children, with a growing number of severe and even fatal cases reported in both children and immunocompetent adults^8-10^. The study by Zhong *et al*.^11^ shows that severe adenovirus pneumonia (SAP) accounts for about 3.01% of hospitalized pediatric pneumonia cases. More than 80% of adenovirus pneumonia cases occur in children aged under four years, especially in infants and young children <2 years old^12^, who are most susceptible to ADV infection, often in combination with other pathogens or multiple infections. The incubation period for ADV generally ranges from 2 to 21 days, with a peak contagious period occurring from the end of incubation to the acute phase. Clinical manifestations of ADV infection vary widely, influenced by factors such as age, viral strain, underlying disease, and immune status^5^. Children under two years old, those with chronic cardiopulmonary diseases, malnutrition, and weakened immune systems are at high risk of severe ADV pneumonia^13-15^. The emergence of variant strains has contributed to a rise in severe and life-threatening cases, emphasizing the need for enhanced care. Mechanical ventilation is often necessary for patients experiencing respiratory failure or acute respiratory distress syndrome. A crucial aspect of clinical research focuses on optimizing weaning strategies and minimizing ventilator-associated complications^16,17^. In recent years, evidence-based nursing has emerged as a valuable approach in critical care settings. By integrating the best clinical evidence into practical experience, evidence-based care is a robust framework for developing effective treatment strategies. However, its application to ventilator treatment for children with severe adenovirus pneumonia remains relatively unexplored in the current literature^18,19^.

Therefore, ensuring the safety of ventilator treatment needs proactive clinical care practices. Evidence-based nursing methods offer a systematic approach to identifying reliable nursing measures. By analyzing patient cases, identifying risk factors, and seeking relevant evidence-based support, healthcare providers can develop tailored nursing strategies to optimize current care programs. This study aimed to evaluate the impact of evidence-based nursing care on improving clinical outcomes in mechanically ventilated children with adenoviral pneumonia. Additionally, this study examined the effects of this approach on the length of hospital stay, duration of mechanical ventilation, time to symptom resolution, and complication rates.

## MATERIALS AND METHODS

### Study design and setting

This prospective randomized controlled trial was conducted on children with ADV pneumonia admitted to the Hunan Provincial People’s Hospital (the First Affiliated Hospital of Hunan Normal University) in Changsha from February 2018 to December 2021.

### Ethics

Written consent was obtained from patients who fully understood the content of the study. This study was approved by the Research Ethics Committee of Hunan Provincial People’s Hospital (Nº 2023-224).

### Participants and sampling

Inclusion criteria: 1) Children diagnosed with severe ADV pneumonia (as defined by the Guidelines for the Treatment of Adenovirus Infections^20^); 2) Children under 14 years old diagnosed with ADV infection; 3) Positive venous blood adenovirus IgM test and positive respiratory adenovirus test; 4) Lung patchy shadows on chest radiography and arterial partial pressure of oxygen (PaO2) ≤ 60 mmHg or PaCO2 ≥ 50 mmHg before mechanical ventilation; 5) Mechanical ventilation exceeding 48 hours; 6) Complete clinical data available for analysis, and 7) Informed consent obtained and signed by the child’s family.

Exclusion criteria: 1) Children with malignant tumors, hematological or immune system diseases; 2) Children with mental disorders; 3) Children with severe liver and kidney dysfunction; 4) Children with congenital heart disease, and 5) Children with coinfections in other organs or systems.

**Figure 1:**
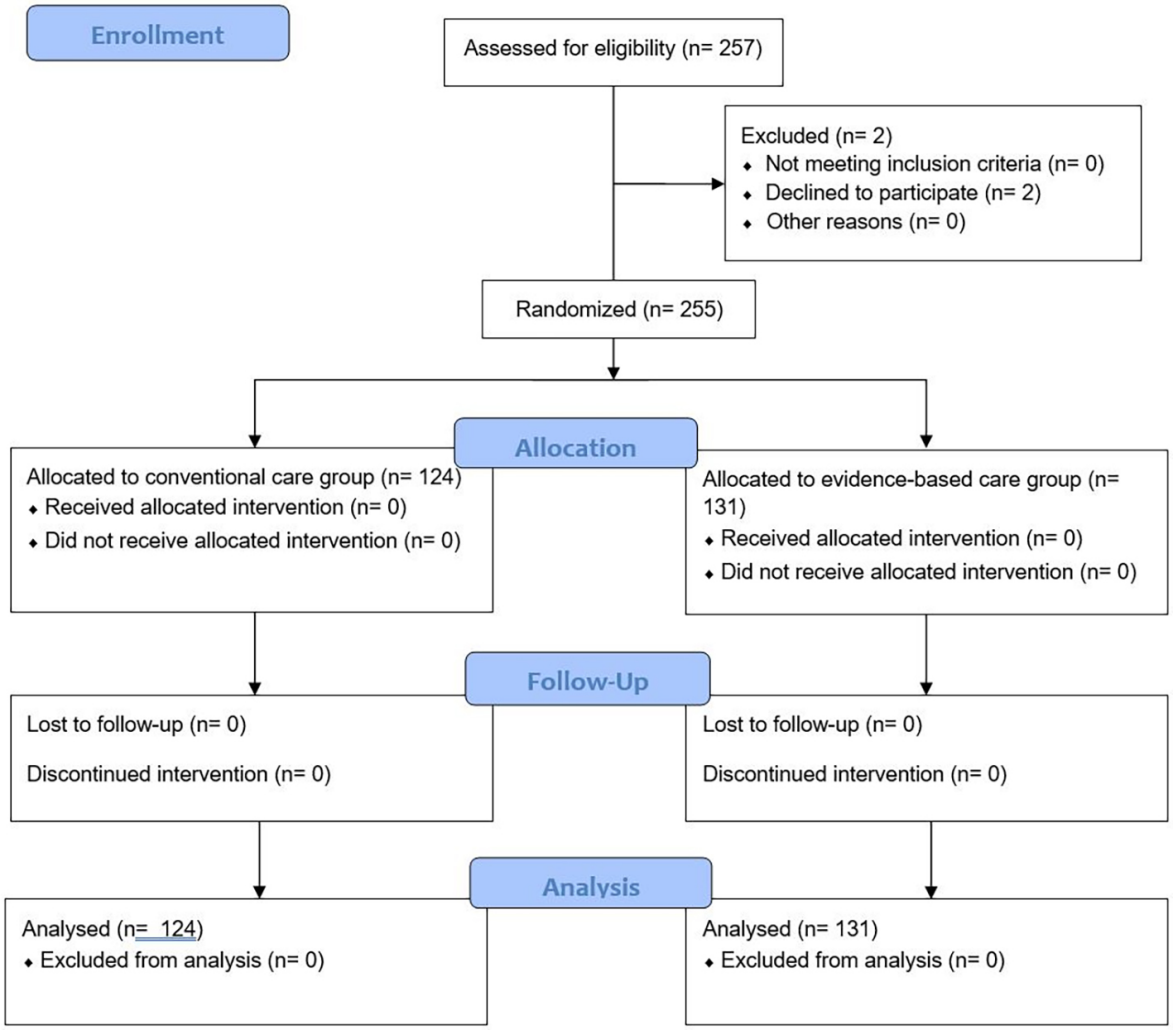
Study flowchart.

A total of 257 children with ADV pneumonia were assessed for eligibility criteria. Two patients withdrew from the study, leaving 255 patients. These participants were divided into two groups according to the random number table, including 124 cases in the conventional care group and 131 cases in the evidence-based care group (Figure 1).

### Intervention

The conventional care group received routine care, which included the following:

1. Environmental care: isolation, temperature and humidity control, regular ventilation and disinfection, and a quiet environment.
2. Dietary care: high-protein, vitamin-rich liquid or semi-liquid feedings through nasal tube, limited to 200 mL per feeding for all ages.
3. Respiratory care: nebulization treatment, chest physical therapy, nasal and oral suctioning, oxygen therapy, appropriate positioning for ventilated patients, and prevention of gastric reflux.
4. Vital sign monitoring: close monitoring of vital signs with prompt notification of any abnormalities to the physician.
5. Psychological care: use of understandable body language, toys, or snacks to reduce anxiety and fear, and support for the child and family.
6. Health education: explanation of ADV pneumonia, treatment guidelines, prevention strategies (avoiding contact with sick children, hand hygiene, and maintaining appropriate warmth).

Children in the intervention group received evidence-based nursing care, implemented by a specialized team consisting of a nurse manager, a charge nurse, and a resident. The team members underwent training and assessment to ensure adherence to evidence-based practices. Key factors and risks associated with airway care during ventilator treatment were identified via literature review, clinical experience, and expert input. These factors included:

1. Airway assessment: regular assessment of airway status, identification of risk factors for tracheal tube dislocation, and implementation of appropriate preventive measures (e.g., racket-type restraint belts, continuous observation, and clear communication).
2. Positioning: optimal positioning to facilitate ventilation, including head-of-bed elevation and prone positioning.
3. Airway humidification: maintenance of airway moisture using appropriate humidification measures, nebulized medications, and saline solution selection.
4. Oral care: regular oral care with 2% soda solution to prevent oral infections and facilitate secretion clearance.
5. Suctioning timing and technique: timely and effective suctioning based on clinical assessment, using appropriate techniques to minimize trauma and optimize oxygenation.
6. Endotracheal tube cuff pressure: maintenance of appropriate cuff pressure to prevent airway damage and aspiration.
7. Infection prevention and control: strict adherence to disinfection and isolation protocols, including contact and droplet precautions, hand hygiene, and environmental cleaning.
8. Psychological support: provision of psychological support for both children and families to alleviate anxiety and promote coping mechanisms. Regular evaluation of the evidence-based care program was conducted to identify improvement areas and optimize patient outcomes.

### Measures

Daily progress was monitored for all children in both groups, including the resolution time of main signs and symptoms (cough, sputum, pulmonary rales, and body temperature), length of hospital stays, complication rates, and blood biomarker levels. The total effective rate was calculated as follows: (number of discharged patients + number of improved patients)/total number of patients × 100%. The double-blind assessment was conducted by three evaluators.

### Evaluation criteria

1. Length of Hospital stay: the length of hospital stay was recorded as a measure of therapeutic efficacy.
2. Mechanical ventilation duration: the number of days requiring mechanical ventilation was recorded to assess the impact of the interventions.
3. Clinical symptom improvement time: the rate of improvement in cough, sputum, pulmonary rales, and fever was measured.
4. Complication incidence rate: the occurrence of ventilator-associated complications and other adverse events (such as myocardial injury, respiratory failure, heart failure, and liver damage) was recorded and compared between groups.
5. Ventilation and weaning success rate: the percentage of patients successfully weaned off mechanical ventilation was recorded and analyzed.
6. Blood biochemical indicators: levels of leukocytes, procalcitonin (PCT), and C-reactive protein (CRP) were measured to assess the inflammatory response and infection status before and after intervention. Complete blood count was done using a Cell-Dyne 1600 System (Abbott Park Laboratories, Ilinois, USA). C-reactive protein (CRP) was measured using a latex agglutination assay on a Vitro 950 analyzer (Johnson & Johnson, Rochester, NY, USA). Procalcitonin was measured using an immunoluminometric assay Behring Diagnostics analyzer (Marburg, Germany). Interassay and intraassay variability were < 7% and 7%, respectively. At room temperature, the stability of PCT can be observed regularly in blood samples and be measured together with routine variables. The results of Serum PCT can be obtained in 2 h and only 20 µl of serum is needed^21^.

### Statistical analysis

Data analysis was performed using SPSS 20.3. Statistical data were expressed as the number of cases and percentages (%) using the χ2 test. The data were expressed as mean ± standard deviation and t-test. P < 0.05 was considered statistically significant.

## RESULTS

The conventional care group consisted of 85 males and 39 females, aged from 10 to 106.4 months and a mean age of 54.2±12.8 months. The evidence-based care group included 90 males and 41 females, with ages ranging from 10.3 to 106.7 months, with a mean age of 57.9±14.6 months. There were no significant differences in gender and age between the two groups (P > 0.05).

The results showed that in the conventional care group, one patient was discharged, 118 patients showed improvement, two showed no effect, and three cases died. In contrast, in the evidence-based care group, four patients were discharged, 125 patients showed improvement, zero cases showed no effect, and two cases died. The evidence-based care group demonstrated a 98.47% overall effectiveness rate, which was significantly higher than that of the conventional care group (95.97%) (p<0.05). Table 1 indicates that evidence-based care improved patient outcomes.

**Table 1:** Efficiency of care.

Group	Case	Discharge from the hospital	Improvements	No effect	Death	Total efficiency (%)
Conventional care group	124	1	118	2	3	119 (95.97)
Evidence-based care group	131	4	125	0	2	129 (98.47)
χ^2^						1.4979
p						0.221

The results revealed that the evidence-based care group demonstrated a significantly shorter resolution time of cough and sputum, disappearance of pulmonary rales, and body temperature recovery compared to the conventional care group (p<0.05, Table 2).

**Table 2:** Time to the disappearance of major signs and symptoms.

Group	Case	Time for cough and sputum to disappear (Day)	Time to resolution of pulmonary rales (Day)	Body temperature recovery time (Day)
Conventional care group	124	14.76±2.23	12.46±1.83	9.78±1.40
Evidence-based care group	131	12.05±1.85	10.14±1.56	8.32±0.94
χ^2^		5.401	5.338	8.253
p		0.036	0.014	0.045

The results showed that the length of hospital stay and the duration of cough and mechanical ventilation were significantly lower in the evidence-based group compared to the conventional care group (p<0.05). Table 3 shows that evidence-based care significantly reduced the length of hospital stay and the duration of mechanical ventilation in the children.

**Table 3:** Length of hospitalization and duration of mechanical ventilation.

Group	Case	Hospitalization time (Day)	Mechanical ventilation time (days)
Conventional care group	124	26.38±13.21	12.28±2.33
Evidence-based care group	131	16.43±9.47	8.43±3.79
χ^2^		13.401	4.533
p		0.011	0.026

The results showed that the conventional care group had 12 patients with myocardial damage, 11 patients with abnormal liver function, two patients with respiratory failure, two patients with heart failure, two patients with pericardial effusion and one patient with infectious toxic encephalopathy, while the evidence-based care group had six patients with myocardial damage, four patients with abnormal liver function, one patient with respiratory failure, one patient with heart failure, zero patients with pericardial effusion, and zero patients with infectious toxic encephalopathy. The complication rate in the evidence-based care group was 9.16%, which was significantly lower than the overall effective rate of the conventional care group (25.00%) (p<0.05). Table 4 indicates the efficacy of evidence-based care in reducing patient complications.

**Table 4:** ADV pneumonia complications.

Group	Case	Myocardial damage (cases)	Abnormal liver function (cases)	Respiratory failure (cases)	Heart failure (cases)	Pericardial effusion (cases)	Infection with toxic encephalopathy (cases)	Total incidence (%)
Conventional care group	124	12	11	3	2	2	1	31/(25.00%)
Evidence-based care group	131	6	4	1	1	0	0	12/(9.16%)
χ^2^								5.427
p								0.029

The results showed that patients in the evidence-based care group showed significantly lower levels of leukocyte, calcitonin, and C-reactive protein compared to the conventional care group (p<0.05, Table 5). This underscores the significant reduction in leukocyte, calcitonin, and C-reactive protein levels in children under evidence-based care.

**Table 5:** Results of blood biochemical indicators after the intervention.

Group	Case	Leukocyte levels (10^9^/L)	Calcitonin levels (ng/mL)	C-reactive protein levels (mg/L)
Conventional care group	124	7.25±2.9	2.48±3.27	58.7±12.6
Evidence-based care group	131	5.13±3.2	0.98±2.91	37.6±13.1
χ^2^		3.964	8.745	6.890
p		0.034	0.008	0.019

## DISCUSSION

Adenovirus (ADV) is a common cause of acute respiratory infections globally, particularly in children, including in China^22-24^. Due to their underdeveloped respiratory tracts and weaker immune systems, children are at higher risk for severe adenovirus pneumonia, which can lead to high morbidity and mortality rates^25^. ADV infection can affect multiple systems, often manifesting with symptoms like acute fever^26^, dyspnea, cough^27^ and, in severe cases, toxic encephalopathy^28^. To address this challenge, active, effective, and careful symptomatic care measures are crucial to relieve symptoms and improve patient outcomes. Some children may require mechanical ventilation to support their breathing. However, prolonged ventilation can lead to complications and challenges during weaning from the ventilator. These challenges include increased risk of extubation failure, longer periods under ventilation, longer hospital stays, and increased use of healthcare resources. Therefore, careful monitoring of the mechanical ventilation process is crucial for optimizing patient care and minimizing complications.

This study incorporated eight key evidence-based care principles: airway assessment, positioning management, airway humidification, oral care, timely and effective airway suctioning, mask care, sterile isolation measures, and psychological and family support. Compared to conventional care, implementing evidence-based nursing practices can bridge knowledge and skill gaps among nursing staff, enhancing critical thinking and practices. Integrating evidence-based principles into healthcare services can improve the quality of care and empower nursing staff to provide optimal patient care. Evidence-based nursing^29,30^, a relatively new concept in the nursing field, aligns with evidence-based medicine. By combining scientific evidence, clinical experience, and patient needs, nurses can make informed decisions that improve patient outcomes^31-33^. Evidence-based nursing interventions can reduce the length of hospital stays and improve treatment quality for patients with heart failure^34^.

The intervention group exhibited a significantly faster main symptoms resolution (cough, pulmonary rales, and fever) compared to the control group. Key interventions such as positioning management, airway humidification, and airway suction contributed to improved symptom resolution by reducing sputum accumulation, enhancing respiratory tract moisture, and facilitating sputum clearance.

The nursing process emphasized disinfection, isolation measures, and standard prevention practices. Strict adherence to infection prevention protocols during procedures like suctioning and ventilator use, along with rigorous hand hygiene, reduced the risk of healthcare-associated infections. Psychological support and family involvement were central in addressing the emotional distress experienced by children and their families in the hospital setting. By providing emotional support and effective communication, the study aimed to alleviate anxiety, increase patient satisfaction, and enhance cooperation during mechanical ventilation.

The intervention group experienced significantly shorter hospital stays and mechanical ventilation durations compared to the control group. Evidence-based care practices, including oral care, ventilator line intervention with 2% soda, and proper positioning, helped prevent complications like ventilator-associated pneumonia, and reduced the duration of ventilation. A comparative analysis of blood gas indicators further supports the positive effect of evidence-based nursing interventions. However, the inclusion of all children in the region may have introduced potential biases in the study. A more in-depth analysis of factors influencing successful ventilator weaning was not conducted. Additionally, due to the young age of the children, their families mostly assessed subjective indicators. Future research should involve multicenter, prospective randomized controlled clinical trials to provide more robust evidence and insights into the effectiveness of evidence-based nursing interventions in pediatric populations.

This study investigated the weaning process for children with severe ADV pneumonia who required mechanical ventilation. The results showed that the use of an evidence-based care model significantly improved the weaning success rate, laying a foundation for effective clinical care programs. By integrating evidence-based nursing measures and clinical experience, standardized protocols for the management of adenovirus pneumonia can be developed. This approach can lead to improved patient outcomes, including reduced pain, accelerated recovery, shorter hospital stays, and enhanced patient satisfaction.

The results of this study highlighted the significant benefits of evidence-based nursing care in improving clinical outcomes for children with adenoviral pneumonia. By implementing evidence-based care as a standard approach in intensive care units, hospitals can reduce the length of hospital stay and the need for mechanical ventilation, decrease treatment costs, and enhance patient satisfaction. Moreover, the study underscored the importance of training nurses and healthcare teams on evidence-based care to improve the quality of patient care. It is recommended that more educational programs be conducted to enhance the skills of nurses in this area.

### Limitations

The study had several limitations that may impact the generalizability and robustness of the findings. Firstly, the single-center design limited the generalizability of the results to other healthcare settings or patient populations. It is uncertain whether similar outcomes could be observed in different clinical environments or regions with varying levels of healthcare infrastructure. Secondly, the relatively short follow-up period, which ended at hospital discharge, prevented the assessment of long-term outcomes and potential late complications, particularly in pediatric patients with severe adenoviral pneumonia. Thirdly, the study did not account for several potential confounding factors, such as disease severity, comorbidities, and variations in treatment protocols. These unmeasured variables might have influenced outcomes, making it difficult to attribute the observed benefits solely to the evidence-based nursing interventions. To address these limitations, future research should involve multicenter studies with long-term follow-up periods and rigorous control of confounding factors. This will help validate and enhance the reliability of the results.

## CONCLUSION

Evidence-based nursing interventions could improve treatment outcomes, accelerate recovery, and reduce complications in children with ADV pneumonia, warranting their clinical promotion and use.
