# Erratum

**DOI:** 10.1590/S1678-9946202567013err

**Published:** 2025-04-04

On the article "*Evaluating the effects of evidence-based nursing on length of hospital stay, duration of mechanical ventilation, symptom relief, and complication rates in children with severe adenoviral pneumonia: a prospective randomized controlled trial*", published in the Revista do Instituto de Medicina Tropical de São Paulo, Vol. 67, e13, 2025 (https://doi.org/10.1590/S1678-9946202567013):

Where it reads:

Hunan Normal University, Hunan Provincial People's Hospital

Should be read":

Hunan Provincial People's Hospital (The First Affiliated Hospital of Hunan Normal University)

